# Improving the Strapdown Airborne Vector Gravimetry by a Backward Inertial Navigation Algorithm

**DOI:** 10.3390/s18124432

**Published:** 2018-12-14

**Authors:** Minghao Wang, Juliang Cao, Shaokun Cai, Meiping Wu, Kaidong Zhang, Ruihang Yu

**Affiliations:** National University of Defense Technology, Changsha 410073, China; wang900304@nudt.edu.cn (M.W.); shaokuncai@nudt.edu.cn (S.C.); meipingwu@263.net (M.W.); zhangkaidong@nudt.edu.cn (K.Z.); yuruihang@nudt.edu.cn (R.Y.)

**Keywords:** strapdown vector gravimetry, backward inertial navigation algorithm, forward backward fusion, SGA-WZ02

## Abstract

Strapdown airborne gravimetry is an efficient way to obtain gravity field data. A new method has been developed to improve the accuracy of airborne vector gravimetry. The method introduces a backward strapdown navigation algorithm into the strapdown gravimetry, which is the reverse process of forward algorithm. Compared with the forward algorithm, the backward algorithm has the same performance in the condition of no sensor error, but has different error characteristics in actual conditions. The differences of the two algorithms in the strapdown gravimetry data processing are presented by simulations, which show that the two algorithms have different performance in the horizontal attitude measurement and convergence of integrated navigation filter. On the basis of detailed analysis, the procedures of accuracy improvement method are presented. The result of this method is very promising when applying to an actual flight test carried out by a SGA-WZ02 strapdown gravimeter. After applying the proposed method, the repeatability of two gravity disturbance horizontal components were 1.83 mGal and 1.80 mGal under the resolution of 6 km, which validate the effectiveness of the method. Furthermore, the wavenumber correlation filter is also discussed as an alternative data fusion method.

## 1. Introduction

The information of Earth’s gravity field is important in geophysics, geodesy, resource exploration, etc. [1]. In order to accurately determine the information of the gravity field, there are several methods, such as satellite gravimetry, airborne gravimetry, shipborne gravimetry and ground gravimetry [2]. The satellite gravimetry can achieve the task of determination high precision global gravity field, but it cannot obtain the gravity information in the high frequency band. The ground gravimetry is the initial and the most classical method to survey the gravity field, the shortcoming of which is inefficiency and confined to the terrain. Shipborne gravimetry, just as its name, uses a ship to measure the gravity field information. Among all the gravity measurement methods, airborne gravimetry is an effective method to collect high precision and high resolution large areas gravity field datum [3].

There are different types of airborne gravimeters to achieve the task of airborne gravimetry and noted that the different principles are used in different airborne gravimetry systems. This means that the stable platform technology is used in GT series gravimeters, AIRGrav gravimeter and Cheken–AM gravity system [4,5], while a Strapdown Inertial Navigation System (SINS) is used in SISG and SGA-WZ [6,7]. Furthermore, the airborne gravimeter can be divided into scalar gravimeter and vector gravimeter according to the target of measurement. The GT gravimeter and Cheken–AM gravity system are scalar gravimeters, while the AIRGrav gravimeter, SISG and SGA-WZ can obtain the gravity vector information.

The platform gravimeter was firstly used in the gravimetry survey. The GT gravimeter was developed by the GT company (Moscow, Russia) and completed more than 200,000 kilometers scalar gravimetry task all over the world, with a precision of 0.6 mGal (mGal ≈ 10^−5^ m/s^2^) under the resolution of 3 km [8]. The AIRGrav gravimeter can measure the three components of gravity field aided by Canadian Gravimetric Geoid 2005 (CGG05) [9]. The repeatability of vertical components can reach the 0.2 mGal and the repeatability of horizontal was about 2 mGal [9].

With the breakthroughs of inertial technology, the research on strapdown gravimetry stemmed from the 1990s. Using a math platform instead of physical platform, the strapdown gravimeter has a lower cost, smaller size and simpler structure than the platform gravimeters. In addition, the strapdown gravimeter can directly measure the gravity disturbance vector. The first airborne gravimeter, SISG, was developed by the University of Calgary [7]. The presented results showed that accuracies of approximately 4 mGal and 6 mGal can be achieved for the vertical and horizontal gravity components by taking advantage of a new algorithm in the inertial frame and wavenumber coefficient filter (WCF) [10]. Moreover, the navigation-grade SINS could be regarded as a strapdown gravimeter and show 0.5–3.2 mGal precision in scalar gravimetry [11]. Through thermal calibration and correction, the accuracy of navigation-grade SINS was superior to 2 mGal when used as a gravimeter [12]. The SGA-WZ series gravimeter is the strapdown gravimeter developed by a National University of Defense Technology. After optimizing the stability and the environment adaptability, the vertical gravity disturbance accuracy new generation of SGA-WZ gravimeter is better than 1 mGal under the resolution of 3 km [13]. In addition, the France Aerospace Lab has developed an absolute shipborne gravimeter and the precision is superior to 1 mGal [14].

The scalar gravimetry is almost mature, the accuracy of which can satisfy the requirements of most applications at present [15]. However, the vector gravimetry still has a great potential in the accuracy promotion for researchers to strive. For example, wavelet decomposition was used to decline the error in the vector gravimetry and the accuracy was about 7 mGal [16]. In addition, the artificial neural network was also applied into vector gravimetry [17]. After algorithm optimization, the repeatability of the data collected by Calgary could directly reach 4–8 mGal in three directions, which was improved to 2–4 mGal after applying WCF [18]. To reduce the negative effect of the gravity vector itself on the measurement, the iterative method was presented in the different research and showed promising results [19]. The high precision gravity model, for example EGM2008, was introduced into vector gravimetry to correct the low frequency error [20].

WCF is a widely used method to eliminate the measurement error in gravimetry and former research has shown its excellent performance. However, the application of WCF needs at least two repeated lines and an empirical threshold, which means low efficiency and restrictions. In this study, the backward strapdown inertial navigation algorithm was introduced into vector gravimetry. The backward algorithm was widely used in the fast initial alignment of autonomous underwater vehicle navigation [21]. When compared with a forward strapdown inertial navigation algorithm, the backward algorithm has the same performance in an ideal condition and different error characteristics in actual sensor error conditions [22]. The prerequisite of repeat lines in WCF is not needed because the data could be processed separately by the forward and backward algorithm. Based on the different characteristics, the weighted equation from optimal linear smoothing could be taken into application to fuse the results from the forward algorithm and backward algorithm [23].

The rest of this paper is organized as follows. Section 2 introduces the backward strapdown inertial navigation algorithm and the principle of strapdown gravimetry. Both the simulations about the different characteristics of two algorithms and the proposed accuracy improvement method are presented in Section 3. An airborne gravimetry flight test using SGA-WZ02 is used to elaborate the actual performance of the method in Section 4. Section 5 discusses possible situations in the application of the method, which include using WCF instead of the variance weighted equation, the performance under different resolutions and the absence of gravity field model information. Finally, conclusions are drawn in Section 6.

## 2. Backward Strapdown Inertial Navigation Algorithm and the Method of Strapdown Vector Gravimetry

### 2.1. Backward Strapdown Inertial Navigation Algorithm

Backward strapdown inertial navigation algorithm is the reverse process of forward (normal) strapdown inertial navigation algorithm. First of all, the primary reference coordinate frames in the strapdown inertial navigation are summarized as follows:

*i*-frame: inertial frame in the law of Newton,
*e*-frame: earth-centered Earth-fixed (ECEF) reference frame,
*n*-frame: local geographic navigation frame (North-East-Down, NED),
*b*-frame: inertial sensor unit body frame (Front-Right-Down, FRD).

A strapdown inertial navigation algorithm could be elaborated by the following equations [24]:

(1) $$\begin{array}{cc} {\dot{v}}_{e}^{n} & =C_{b}^{n}f^{b}-\left(2\omega_{ie}^{n}+\omega_{en}^{n}\right)\times V_{e}^{n}+g^{n},\\ {\dot{C}}_{b}^{n} & =C_{b}^{n}\left[\omega_{nb}^{b}\times \right],\\ \omega_{nb}^{b} & =\omega_{ib}^{b}-C_{b}^{n}\left(\omega_{ie}^{n}+\omega_{en}^{n}\right), \end{array}$$

where $v_{e}^{n}$ is the velocity in the *n*-frame, $f^{b}$ is the specific force measured by quartz flex accelerometers in *b*-frame, $C_{b}^{n}$ is the transformation matrix from *b*-frame to *n*-frame, $\omega_{ie}^{n}$ is the angular velocity of the earth respecting to the *n*-frame, $\omega_{en}^{n}$ is the rotation rate of the *n*-frame due to vehicle rate over the ellipsoid, $g^{n}$ is the gravity vector, [⋆×] denotes the skew symmetric matrix of [⋆] and $\omega_{ib}^{b}$ is the output of gyros.

If the initial value of transformation matrix, velocity and position are known, Equation (1) could be used to compute navigation information based on the output of accelerometers and gyros. However, the navigation algorithm is actually a data processing technique on the computer, so the discrete-time forms of the navigation equations should be derived. Supposing the sampling interval of inertial sensors is $T_{s}$, the discrete-time navigation equations could be elaborated as Equation (2):

(2) $$\begin{array}{cc} v_{ek}^{n} & =v_{ek-1}^{n}+T_{s}\left[C_{bk-1}^{n}f_{k}^{b}-\left(2\omega_{iek-1}^{n}+\omega_{enk-1}^{n}\right)\times v_{ek-1}^{n}+g_{k-1}^{n}\right],\\ C_{bk}^{n} & =C_{bk-1}^{n}\left(I+T_{s}\left[\omega_{nbk}^{b}\times \right]\right),\\ \omega_{nbk}^{b} & =\omega_{ibk}^{b}-{\left(C_{bk-1}^{n}\right)}^{T}\left(\omega_{iek-1}^{n}+\omega_{enk-1}^{n}\right), \end{array}$$

where *k* and k−1 are the sampling times.

Suppose the SINS moving from position A at timepoint k−1 to position B at timepoint *k*, and then the backward algorithm from B to A could be derived from Equation (2), which is shown in Equation (3) [22]:

(3) $$\begin{array}{cc} v_{ek-1}^{n} & =v_{ek}^{n}-T_{s}\left[C_{bk-1}^{n}f_{k}^{b}-\left(2\omega_{iek-1}^{n}+\omega_{enk-1}^{n}\right)\times v_{ek-1}^{n}+g_{k-1}^{n}\right],\\ C_{bk-1}^{n} & =C_{bk}^{n}{\left(I+T_{s}\left[\omega_{nbk}^{b}\times \right]\right)}^{-1}\approx C_{bk}^{n}\left(I-T_{s}\left[\omega_{nbk}^{b}\times \right]\right)=C_{bk}^{n}\left(I+T_{s}\left[{\hat{\omega }}_{nbk}^{b}\times \right]\right),\\ {\hat{\omega }}_{nbk}^{b} & =-[\omega_{ibk-1}^{b}-{\left(C_{bk}^{n}\right)}^{T}\left(\omega_{iek}^{n}+\omega_{enk}^{n}\right)]. \end{array}$$

Since the backward navigation algorithm makes uses of the data from the end to the beginning, the relationships between the subscripts in the forward and backward process, for example, using $C_{bk-1}^{n}$, are shown as Equation (4):

(4) $$C_{bk-1}^{n}=C_{bm-p}^{n}={\hat{C}}_{bp}^{n},$$

where k−1 is the sampling point in the forward process, *m* is the total number of sampling and *p* is the sampling point in the backward process.

Based on Equations (3) and (4), the complete backward navigation algorithm could be obtained:

(5) $$\begin{array}{cc} {\hat{v}}_{ep}^{n} & ={\hat{v}}_{ek-1}^{n}+T_{s}\left[{\hat{C}}_{bk-1}^{n}{\hat{f}}_{p}^{b}-\left(2{\hat{\omega }}_{iep-1}^{n}+{\hat{\omega }}_{enp-1}^{n}\right)\times {\hat{v}}_{ep-1}^{n}+g_{p-1}^{n}\right],\\ {\hat{C}}_{bp}^{n} & ={\hat{C}}_{bp-1}^{n}\left(I+T_{s}\left[{\hat{\omega }}_{nbp-1}^{b}\times \right]\right),\\ {\hat{\omega }}_{nbp}^{b} & ={\hat{\omega }}_{ibp-1}^{b}-{\left({\hat{C}}_{bp-1}^{n}\right)}^{T}\left({\hat{\omega }}_{iep-1}^{n}+{\hat{\omega }}_{enp-1}^{n}\right). \end{array}$$

**Figure 1:**
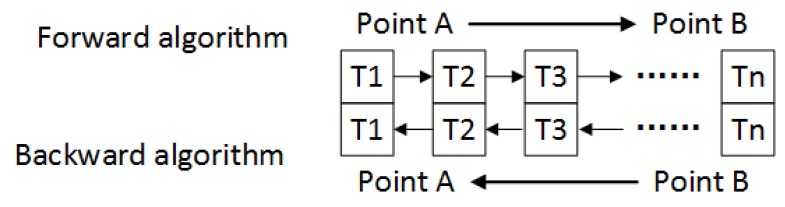
Time sequence in forward and backward algorithms.

Comparing Equations (2) and (5), the backward algorithm has the same structure with the forward algorithm. If the signs of gyro output and earth rotation rate are taken as negative, the backward navigation could be achieved. Figure 1 shows the different time sequence in the forward algorithm and backward algorithm. Without considering sensors’ errors, the attitude and position are the same, but the angular rate and velocity are opposite to each other at the same point in two algorithms. What needs to be pointed out is that the inertial compensation algorithms in forward navigation, for example the coning compensation algorithm, could also be applied in backward navigation. The difference between forward and backward strapdown inertial navigation algorithms in an unideal condition will be shown in Section 3.1.

### 2.2. Method of Strapdown Vector Gravimetry

The principle of strapdown gravimeter in *n*-frame is expressed by Equation (6), which is based on Newton’s equation of motion. Compared with the velocity updating equation in an inertial navigation algorithm, the gravity information is divided into two parts. One part is the normal gravity vector obtained by the reference ellipsoid model, for example WSG84, and the other is the desired gravity disturbance vector. In the principle of strapdown airborne gravimetry, $C_{b}^{n}f^{b}$ is provided by the SINS, while other information comes from global navigation satellite system (GNSS) [6]:

(6) $$dg^{n}={\dot{v}}_{e}^{n}-\left(C_{b}^{n}f^{b}-\left(2\omega_{ie}^{n}+\omega_{en}^{n}\right)\times v_{e}^{n}+\gamma^{n}\right),$$

where $dg^{n}$ is the disturbance gravity vector in *n*-frame and $\gamma^{n}$ is the normal gravity.

The essential error model used in the Kalman filter is shown in Equation (7) [24]:

(7) $$\begin{array}{cc} \delta \dot{p} & =\delta v,\\ \delta \dot{v} & =\left[f^{n}\times \right]+C_{n}^{b}\delta f^{b}-\left(2\omega_{ie}^{n}+\omega_{en}^{n}\right)\times \delta v_{e}^{n}-\left(2\delta \omega_{ie}^{n}+\delta \omega_{en}^{n}\right)\times v_{e}^{n},\\ \dot{\psi } & =-\omega_{in}^{n}\times \psi +\delta \omega_{in}^{n}-C_{b}^{n}\delta \omega_{ib}^{b}, \end{array}$$

where δp, δv and ψ are the position, velocity and attitude errors in *n*-frame.

**Figure 2:**
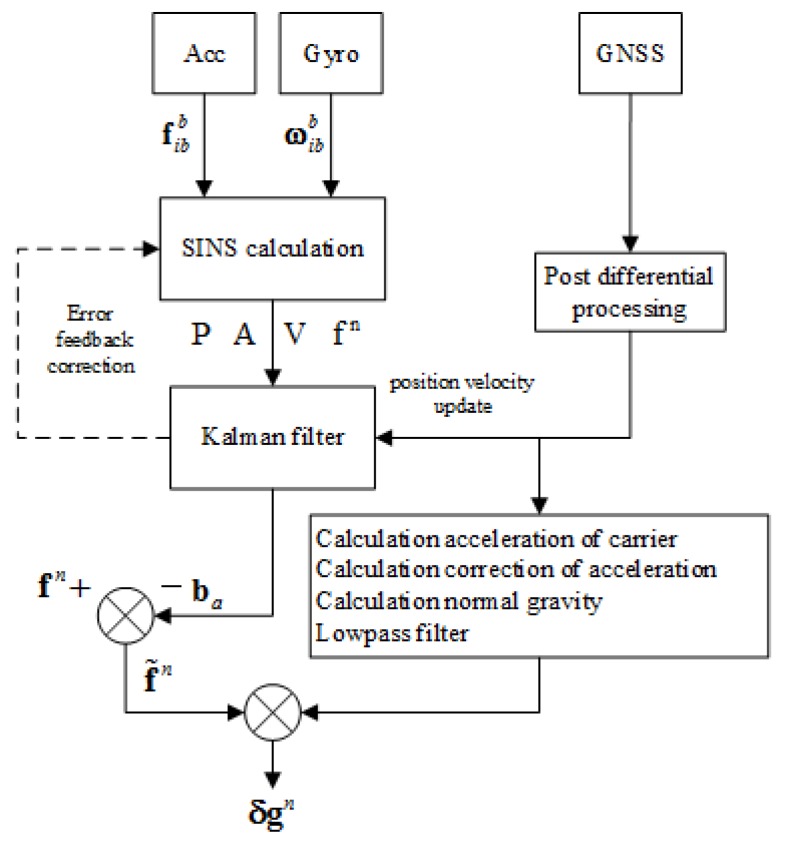
Strapdown airborne gravimeter data processing block diagram.

To insure the accuracy of gravity disturbance determination, the Kalman filter based on position and velocity update is used to estimate the attitudes and the specific force in the airborne gravimetry. The error model in the Kalman filter is shown in Equation (8), in which the bias of accelerometer and gyro are also considered. The observation equation is shown as Equation (9). The flow chart of strapdown airborne gravimeter data processing could be described as Figure 2:

(8) $$\left[\begin{array}{c} \delta \dot{p}\\ \delta \dot{v}\\ \dot{\psi }\\ \dot{b_{a}}\\ \dot{b_{g}} \end{array}\right]=\left[\begin{array}{ccccc} PP & PA & PV & 0 & 0\\ PP & PA & PV & C_{b}^{n} & 0\\ PP & PA & PV & 0 & -C_{b}^{n}\\ 0 & 0 & 0 & 0 & 0\\ 0 & 0 & 0 & 0 & 0 \end{array}\right]\left[\begin{array}{c} \delta p\\ \delta v\\ \psi \\ b_{a}\\ b_{g} \end{array}\right]+\left[\begin{array}{cc} 0 & 0\\ C_{b}^{n} & 0\\ 0 & -C_{b}^{n}\\ 0 & 0\\ 0 & 0 \end{array}\right]\left[\begin{array}{c} \varepsilon_{a}\\ \varepsilon_{g} \end{array}\right],$$

where PP, PV, PA, VP, VV, VA, AP, AV and AA are the error state transfer matrix in the SINS which are the abbreviations of Equation (7) and are shown in the Appendix A [24], $b_{a}$ is the bias of accelerometer, $b_{g}$ is the bias of gyro, $\varepsilon_{a}$ is the white noise of accelerometer and $\varepsilon_{g}$ is the white noise of gyro:

(9) $$Z=\left[\begin{array}{c} \delta p\\ \delta v \end{array}\right]=\left[\begin{array}{c} p_{GNSS}^{n}-p_{SINS}^{n}\\ v_{GNSS}^{n}-v_{SINS}^{n} \end{array}\right],$$

where $p_{GNSS}^{n}$ is the position provided by GNSS, $p_{SINS}^{n}$ is the position provided by SINS, $v_{GNSS}^{n}$ is the velocity provided by GNSS and $v_{SINS}^{n}$ is the velocity provided by SINS.

## 3. Applying Backward Navigation Algorithm to Improve Strapdown Airborne Vector Gravimetry

### 3.1. Different Characteristics between Forward and Backward Inertial Navigation Algorithms in the Strapdown Gravimetry

**Figure 3:**
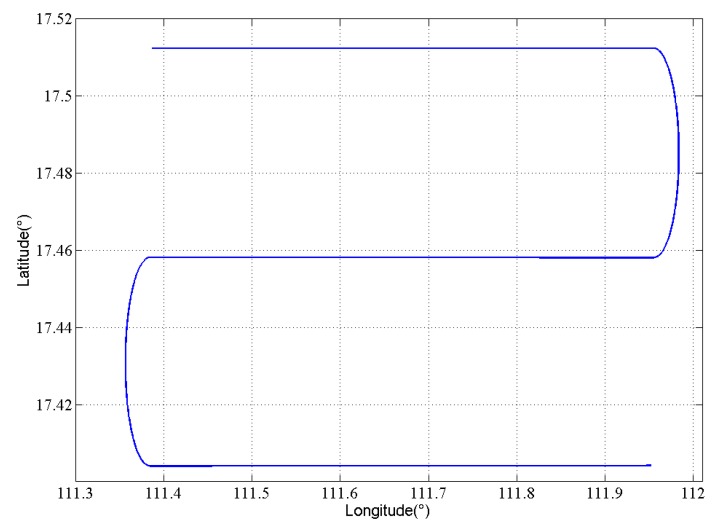
Simulation trajectory.

The critical parts in the strapdown gravimetry is the calculation of inertial navigation and integrated navigation which also contain the main different characteristics between two algorithms. The most important differences between the forward and backward algorithms in airborne gravimetry are the accumulation order of error in standalone inertial navigation calculation and the convergence order of Kalman filter in integrated navigation. The order in forward algorithm is from the starting point to the end point, whereas that in the backward algorithm is from the end point to the starting point. To elaborate on this phenomenon, a simulation experiment is designed. The trajectory of simulation is shown in Figure 3, which contains three straight lines and two turning maneuvers. The length of simulation lines is 60 km and the velocity is 60 m/s, both of which are the typical parameters in the actual airborne gravimetry. The error parameters in simulation are shown in Table 1.

**Figure 4:**
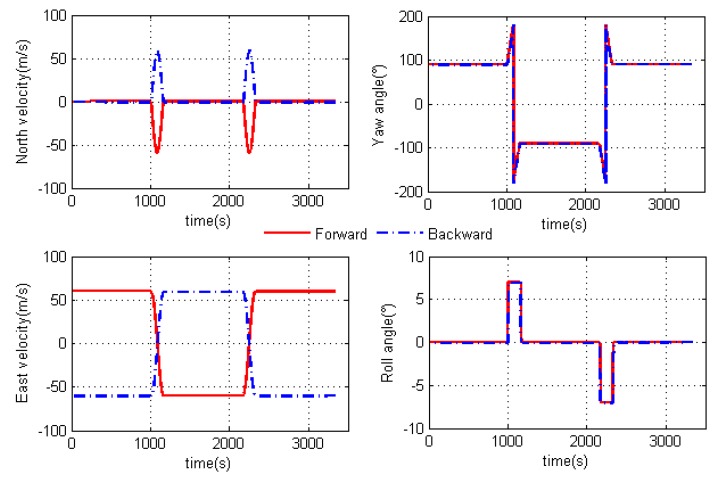
Pure inertial navigation results in two algorithms.

**Figure 5:**
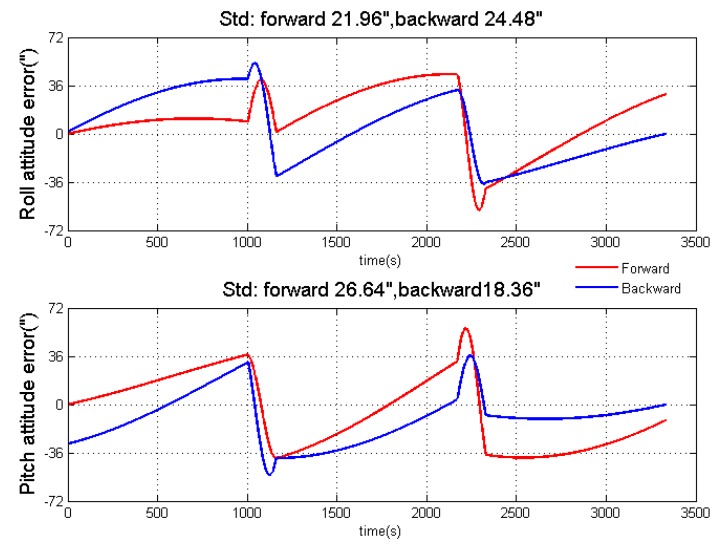
Standalone inertial navigation results after adding errors.

First, the standalone inertial navigation results in two algorithms are shown in Figure 4 under the ideal condition. As mentioned, the velocity of two algorithms are opposite from each other while the attitudes are the same. However, when the error is added in the simulation, the performance of two algorithms is different. Figure 5 is the standalone inertial horizontal attitude error when the error shown in Table 1 is added into the simulation. From Figure 5, we could conclude that the roll angle in forward algorithm has better accuracy than that in the backward algorithm, while the backward algorithm performs better in the calculation of pitch angle.

**Figure 6:**
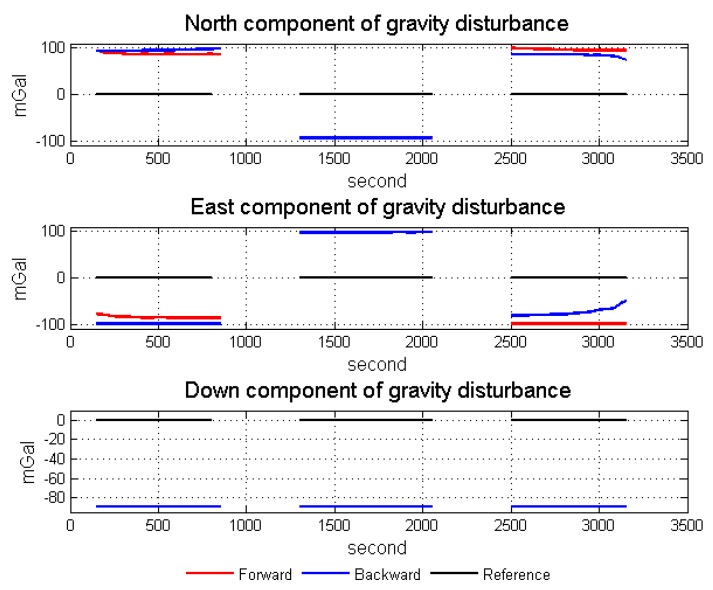
Simulation results of gravity disturbance vectors.

**Figure 7:**
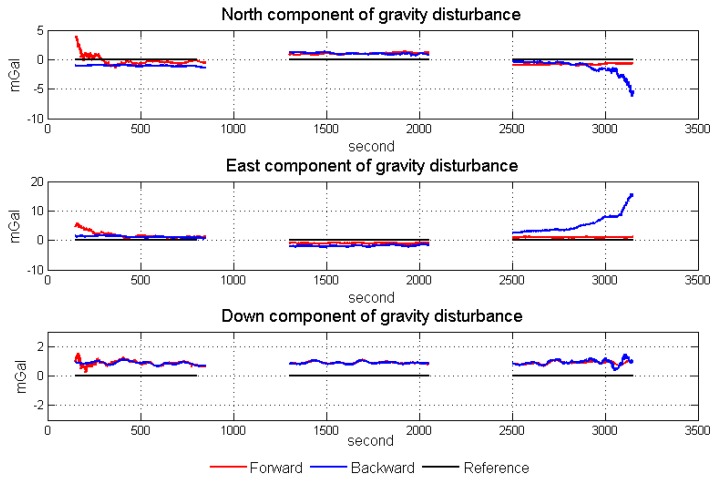
Gravity disturbance vector simulation results after error compensation.

After the analysis of standalone inertial results, the simulation results of gravity disturbance vector along the three simulation survey lines are shown in Figure 6. What needs to be mentioned is that the true value of gravity disturbance vector is set to zero in this simulation experiment. The horizontal components of gravity disturbance are greatly affected by the accelerometer biases when the flight direction changes, while the vertical component suffers less from the accelerometer bias (almost a constant). As mentioned, the accelerometer biases in *b*-frame are estimated by the Kalman filter. Thus, the estimated accelerometer biases after transferring to *n*-frame by direction cosine matrix (DCM) could partly compensate for the measurement error, after which attitude error is the main error affecting the gravimetry. Figure 7 is the simulation results of gravity disturbance vector after accelerometer bias compensation in three lines. The effect of accelerometer bias in gravity disturbance vector results has been reduced much by the compensation. More importantly, the different filtering convergence characteristics between two algorithms have also been shown in Figure 7, especially the horizontal components of gravity disturbance. Both algorithms show better performance in the final part of its data processing sequence due to better observability and convergence.

The two critical parts in airborne gravimetry show different performances when using different algorithms, so there is strong complementarity. Furthermore, the complementarity in horizontal components is much stronger than that in a vertical component. Taking advantage of the complementary characteristics in two algorithms, the accuracy of strapdown vector gravimetry could be improved. In the following section, the methods of forward and backward navigation results fusion are introduced.

### 3.2. Combination of Forward and Backward Navigation Algorithm to Improve the Accuracy of Strapdown Vector Gravimetry

From the simulation analysis in the previous section, we propose a fusion method that can provide beneficial results in vector gravimetry. First, to further combine the data from forward and backward inertial algorithms, the most classical data fusion method is the optimal linear smoothing algorithm. The optimal linear smoothing algorithm uses the observations from the past, the present and the future to obtain an optimal estimation result, so it must be calculated offline, which can be satisfied in the determination of gravity field. The principle of optimal linear smoothing is shown in Equations (10) and (11) [25]:

(10) $$\begin{array}{c} X_{s}=\frac{P_{s}}{P_{f}}X_{f}+\frac{P_{s}}{P_{b}}X_{b}, \end{array}$$

(11) $$\begin{array}{c} P_{s}=\frac{P_{f}*P_{b}}{P_{f}+P_{b}}, \end{array}$$

where $X_{s}$ is the fusion result, $X_{f}$ is the result of forward process, $X_{b}$ is the result of backward process, $P_{s}$ is the fusion variance, $P_{f}$ is the variance of forward process and $P_{b}$ is the variance of backward process

To apply Equation (10) in the forward backward fusion, we should know the variance of gravity disturbance. However, the gravity disturbance is not modeled in the data processing in this paper. Considering the differential form of Equation (6) and only keeping the items related with the SINS, the gravimetry error caused by SINS is shown in Equation (12) [26]. From Equation (12), we can conclude that the vertical component is mainly affected by the accuracy of specific force measurement in the down direction, while the horizontal components are not only influenced by the accuracy of specific force measurement in its own direction but also suffer a lot from the attitude accuracy. One arc second attitude error would induce about 4.8 mGal gravimetry error, so the variance of attitude error in Kalman filter could replace the variance of horizontal gravity disturbance components in the application of the optimal linear smoothing:

(12) $$\delta dg_{sins}^{n}=C_{b}^{n}\delta f^{b}+\Psi \times f^{n}\approx \left[\begin{array}{c} \delta f_{N}-\psi_{E}f_{D}\\ \delta f_{E}+\psi_{N}f_{D}\\ \delta f_{D} \end{array}\right],$$

where $\delta dg_{sins}^{n}$ is the gravity disturbance error vector caused by the SINS, $\delta f^{b}$ is the specific force error in *b*-frame and ψ is the attitude error in *n*-frame.

**Figure 8:**
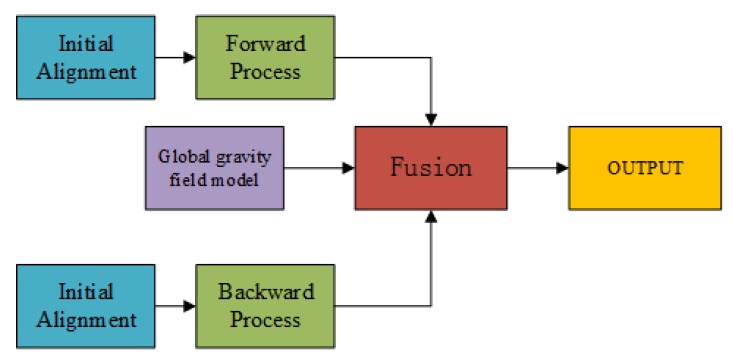
Scheme of forward backward fusion.

We combine the results from forward and backward algorithms to reduce the vector gravimetry error, but another problem to be solved is that the residual accelerometer bias, which could not be estimated by Kalman filter, still affects the vector gravimetry and changes with flight direction. Figure 7 has also shown this phenomenon and the residual accelerometer bias is usually the low frequency error. To conquer this kind of low frequency error, we could fuse the data from the high precision gravity field model [20]. The gravity data from high precision global gravity field model have lower resolution when compared with data from airborne gravimetry, but no low frequency error. The former research has shown that the shortcoming of using a model to correct the error is that the model usually has constant deviation, when it is compared with the truth data in a certain area [27]. However, this shortcoming could be largely eliminated by ground gravimetry and upward extension or by using satellite-only model [28]. Thus, the forward and backward fusion method is divided into four steps, the scheme of which is shown in Figure 8:

1. Use the forward algorithm to get gravity disturbance result,
2. Use the backward algorithm to get gravity disturbance result,
3. Correct the low frequency error by high precision global gravity field model,
4. Fuse the data according to Equations (10) and (11).

Although the weighted equation from classical optimal smoothing is used, the proposed method is different with the classical method. The difference between the classical optimal smoothing and the normal Kalman filter is the use of observations, while the difference between the proposed method and normal Kalman filter is the different characteristics in inertial calculation for the reason of introduction of backward inertial navigation algorithm. The classical optimal smoothing mainly improves the convergence of filtering, while the forward backward fusion in the proposed method not only improves the accuracy of filtering, but also takes advantage of the different characteristics between forward and backward inertial navigation algorithms to improve the accuracy of attitude measurement [29]. In addition, the opposite velocity in the forward process and backward process of the proposed method also leads to different state matrices in data processing, while the backward filter in the classical optimal smoothing uses the state matrix of the forward filter.

**Figure 9:**
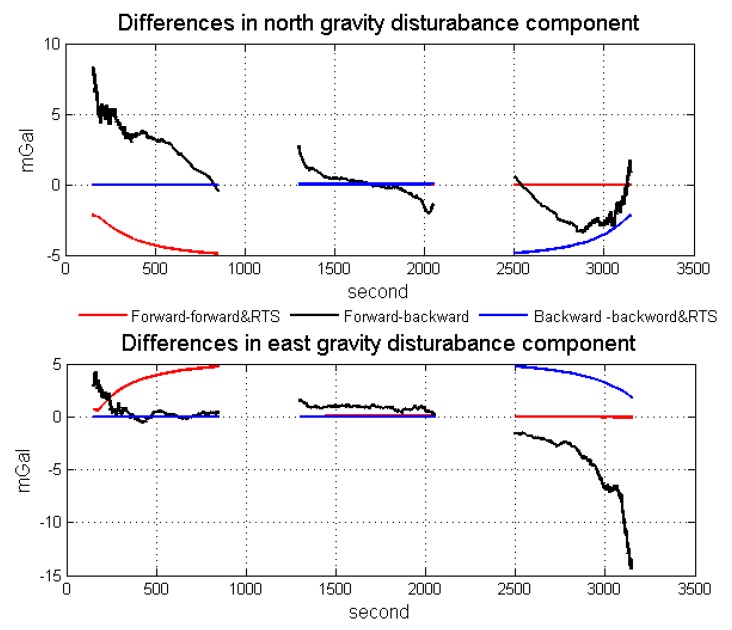
Differences between the forward process, the forward process using classical optimal smoothing, the backward process and the backward process using classical optimal smoothing in the simulation.

Figure 9 is the differences between the forward process, the forward process using classical optimal smoothing, the backward process and the backward process using classical optimal smoothing in the simulation shown in Section 3.1. The red line case represents the difference between the forward process and forward process using optimal smoothing. The main difference lies in the first simulation flight line due to difference in observability and convergence. In the forward process, the first line has weaker observability and poorer filter convergence before any maneuver, but the observability and filter convergence is better in lines 2 and 3 after turning maneuver. In the forward process with optimal smoothing, the three simulation flight lines almost have the same strong observability and good filter convergence due to using all observations. Thus, for the simulation flight line 1, there exist obvious differences, but lines 2 and 3 are almost the same. A similar phenomenon also occurs in the blue line case. However, the phenomenon is different in the black line case (the difference between forward process and backward process), in which all three simulation flight lines show different performances. Thus, the proposed method improves both the accuracy of filtering and the accuracy of integrated navigation.

In the following section, the proposed method is testified by an actual flight test.

## 4. The Actual Improvement in an Airborne Strapdown Gravimetry Test

### 4.1. Test Description

**Figure 10:**
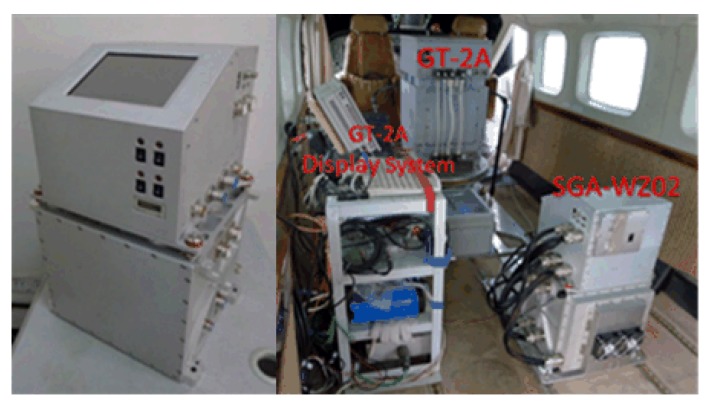
Appearance of SGA-WZ02 and the picture of the flight cabin.

The survey was carried out in the Xinjiang Province of China using SGA-WZ02 in 2015. The gravimetry system was mounted on a Cessna208 aircraft, which was a fixed-wing aircraft with an autopilot. In addition, a GT-2A gravimeter participated in the test. The properties of SGA-WZ02 were shown in Table 2 and the picture of SGA-WZ02 and cabin was shown in Figure 10 [30]. Three GNSS receivers were used for the differential kinematic positioning, one of the receivers was located on the airplane and the others were located on the roof as ground stations. All of the GNSS receivers installed on the ground and on the airplane were NOVATEL OEMV3 (Calgary, AB, Canada). Use WAYPOINT to obtain differential GNSS position result, and then the velocity and acceleration are obtained by the position difference method. Lever arm, which was used to transform the sensitive center of the GNSS receiver to the SINS sensitive center, was ${\left[1.58,-0.1,-1.20\right]}^{\prime }$ m in the *i*-frame. Moreover, the ground vector gravimetry was unavailable in this test, so we regarded the gravity disturbance information at the tarmac as ${\left[0,0,0\right]}^{\prime }$ mGal.

**Figure 11:**
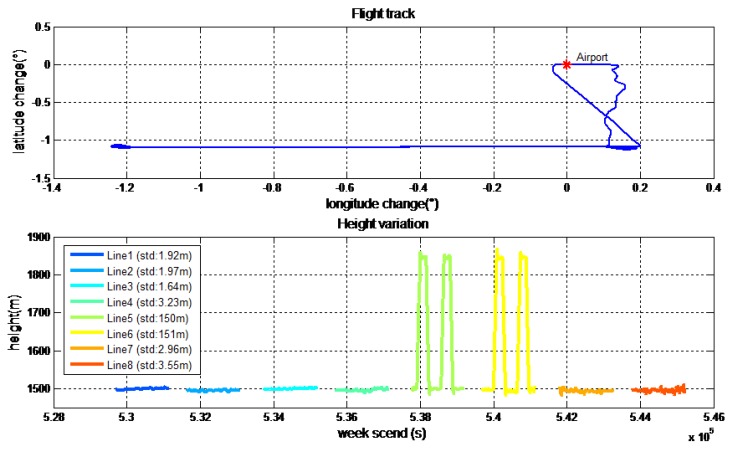
Path of flight test and height variation in lines.

**Figure 12:**
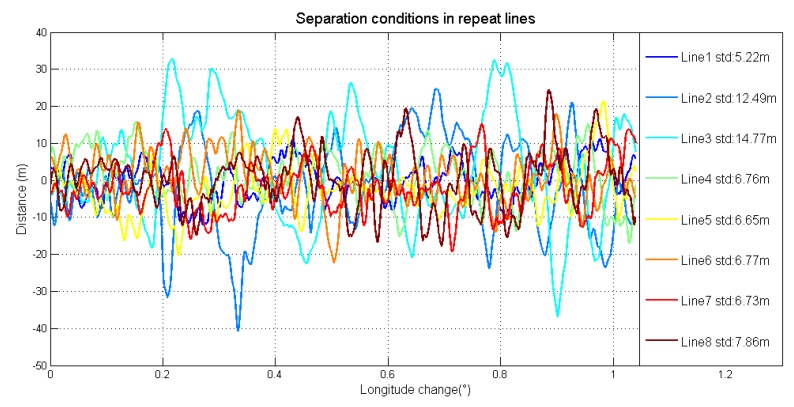
Northward separation conditions in repeat lines.

**Figure 13:**
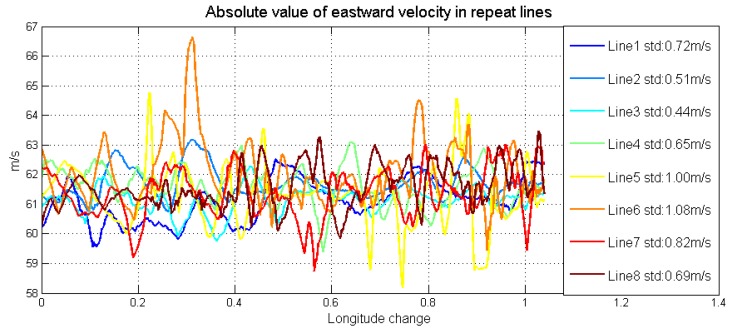
Absolute value of eastward velocity in repeat lines.

The detailed characters of flight were shown in Table 3. Each survey line was about 80 km, whose ends were extended 3 km to eliminate the boundary effect of low-pass filter. In this airborne gravimetry test, the maximum distance between survey area and airport was less than 140 km. Figure 11 is the flight trajectory and height variations in lines. There are eight lines in this flight, among which the fifth and sixth lines are the undulated flight to test the performance of SGA-WZ02 in extremely turbulent conditions. Thus, what we are more concerned about is the result of the normal lines (lines 1–4, 7, 8). The air flow condition was getting worse during the test. Figure 12 is the northward separation conditions and Figure 13 is the absolute value of eastward velocity. The trajectory of lines 2 and 3 have not been well controlled when compared with other lines, and the speed in lines 5 and 6 is not very stable due to undulating flight.

### 4.2. The Improvement Results after the Forward and Backward Algorithm Data Fusion

**Figure 14:**
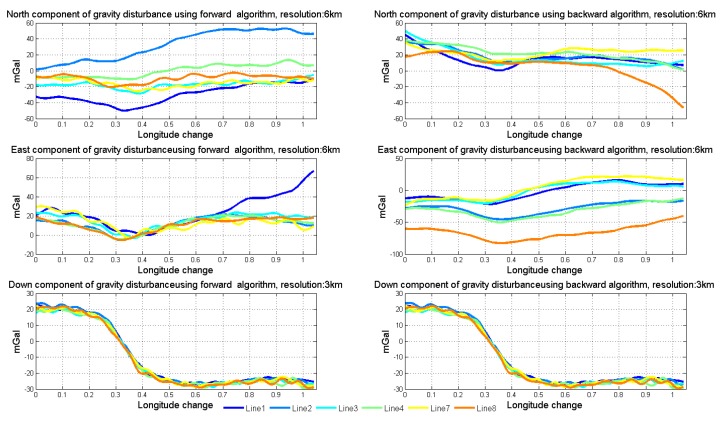
Results of normal lines using two algorithms.

Using the method of strapdown vector gravimetry shown in the former section, we could get the result of airborne gravimetry. During data processing, we use both forward algorithm and backward algorithm to calculate the data collected by SGA-WZ02. The results of normal lines are shown in Figure 14. In order to testify the quality of gravimetry in repeat lines, the repeatability of the gravity disturbance vector can be calculated by Equations (13) and (14) [31]. Equation (13) describes the repeatability of certain lines and Equation (14) is the total repeatability of repeat lines. The repeatability results are shown in Table 4.

**Figure 15:**
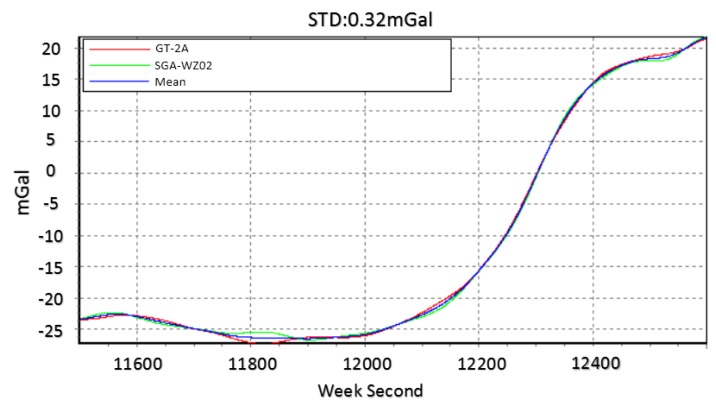
Mean value of vertical component among normal lines in GT-2A and SGA-WZ02.

The down component is in 3 km resolution, while the north and east components are in 6 km resolution. Figure 15 shows the mean values of vertical components among normal lines in SGA-WZ02 and GT-2A gravimeters [30]. The repeatability in a down component is much better than that of north and east components because the attitude error mainly impacts the horizontal of gravity disturbance. Thus, we mainly talked about the horizontal components of gravity disturbance in this paper.

As the simulation points out that the accuracy of north component of gravity disturbance, which is mainly related to the pitch angle error, obtained from the backward algorithm is better than that obtained from the forward algorithm, while the accuracy of the east component of gravity disturbance, which is mainly related to the roll angle error, obtained from the forward algorithm is better than that obtained from the backward algorithm. Obviously, using the north component resulting from the backward algorithm and the east component resulting from the forward algorithm is the simplest way to get better measurement results. However, this way cannot meet the requirements of high accuracy applications and there is a better way to combine the two algorithms.

(13) $$\begin{array}{c} \varepsilon_{j}=\pm \sqrt{\frac{\sum_{i=1}^{n}\delta_{ij}^{2}}{N}},\left(j=1,2,\ldots ,m\right), \end{array}$$

(14) $$\begin{array}{c} \varepsilon =\pm \sqrt{\frac{\sum_{j=1}^{m}\sum_{i=1}^{n}\delta_{ij}^{2}}{N}}, \end{array}$$

where $\varepsilon_{j}$ is the repeatability of repeat line *j*, $\delta_{ij}$ is the different value between the mean value at point *i* and the value in repeat line *j* at point *i*, *N* is the total data point number of common part of repeat lines, *M* is the total line number of repeat line flight, and ε is the repeatability of total repeat line.

Except the difference in the accuracy of two horizontal components, the advantages of two algorithms are the complementarity in the gravimetry. Before fusing the two results, we use EIGEN-6C4, the low frequency part of which only comes from satellite gravimetry to correct the error in low frequency [32]. The method of using gravity model to improve the vector gravimetry has been shown as a reference, in which the result of linear fitting is regarded as the low frequency component and the remaining part as the high frequency component [20]. Equation (15) describes the frequency separation method. Thus, the gravity disturbance can be divided into three parts: bias, linear part and high frequency part, as shown in Equation (16). The bias is obtained from the ground gravimetry and upward extension, the linear coefficient is obtained by gravity field model and the high frequency part is obtained by the data fusion result from the forward algorithm and backward algorithm. The full order EIGEN-6C4 was used to obtain the gravity field model data. Meanwhile, the bias part was also obtained from EIGEN-6C4 because the ground gravity data was unavailable in this flight test:

(15) $$\begin{array}{c} dg_{hf}=dg-dg_{linear}, \end{array}$$

where $dg_{hf}$ is the high frequency part of gravity disturbance, dg is the origin result of gravity disturbance and $dg_{linear}$ is the linear fitting result of gravity disturbance.

(16) $$\begin{array}{c} dg=bias+k_{m}d_{var}+dg_{hf}, \end{array}$$

where dg is the result of gravity disturbance, bias is the bias from the ground gravimetry and upward extension algorithm, $k_{m}$ is the linear coefficient obtained by the gravity field model, $d_{var}$ is the variation of distance and $dg_{hf}$ is the high frequency part of gravity disturbance obtained by the data fusion.

**Figure 16:**
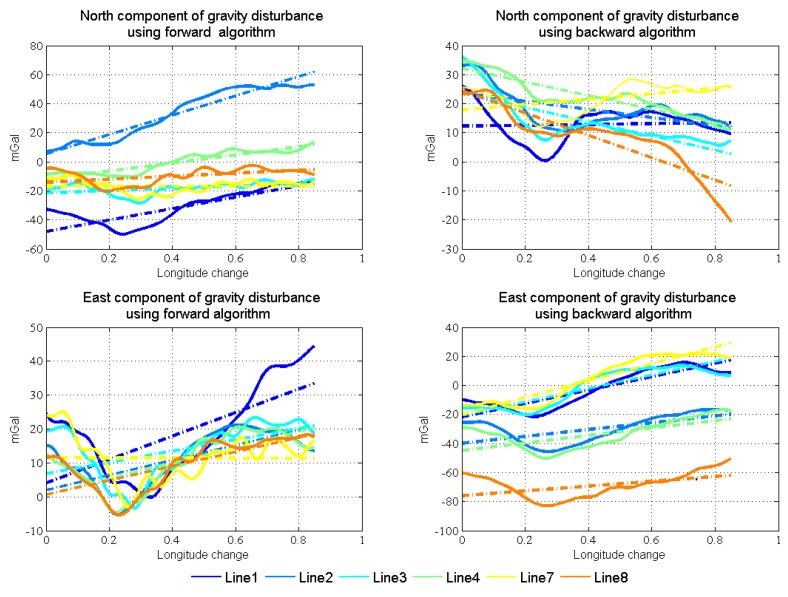
Two horizontal components’ linear fitting results in normal lines.

**Figure 17:**
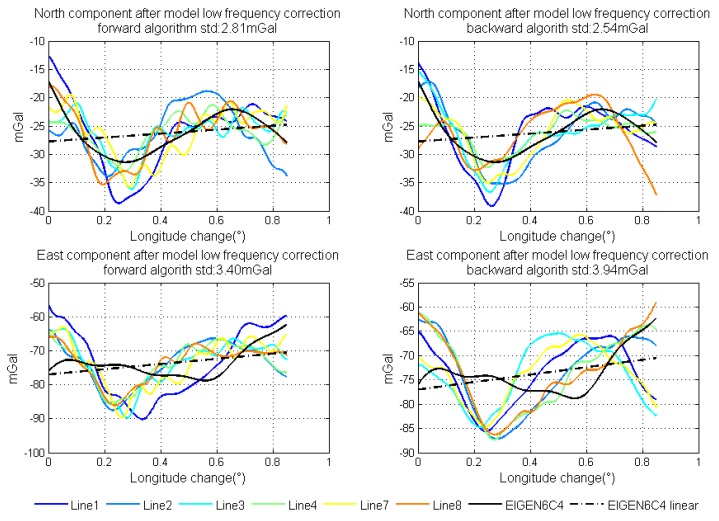
North and east components of gravity disturbance after correction by model.

Figure 16 shows the linear fitting results of the two horizontal components. Figure 17 shows the north and east components of gravity disturbance after correction by the gravity model. After correcting the error in the low frequency band, the repeatability has been promoted a lot in all conditions and the different performances between the two algorithms still exist. The north component obtained from the backward algorithm and the east component obtained from the forward algorithm are better than those obtained from another algorithm. Thus, there is considerable potential for accuracy improvement by fusing the data from two algorithms.

**Figure 18:**
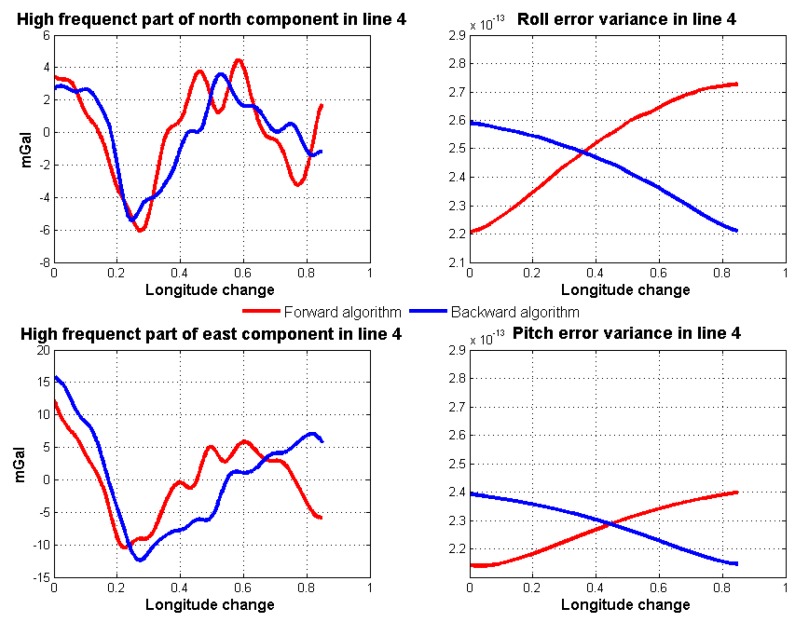
Estimated variances of attitude error and high frequency part of gravity disturbance horizontal components in line 4.

**Figure 19:**
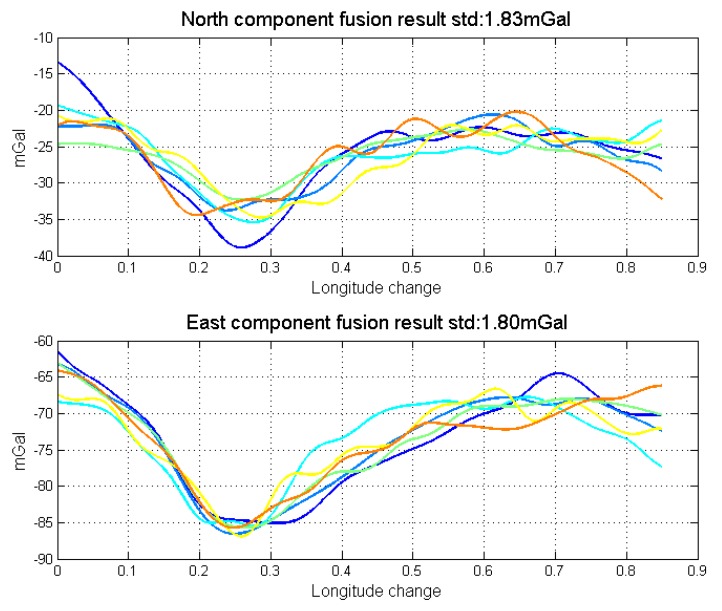
Data fusion results by the accuracy improvement method.

Through gravity model correction, the error in low frequency, especially specific measurement error, has been largely eliminated. The residual error mainly stems from the attitude error. The residual error in the north component was mainly caused by the east attitude (pitch) error, while the residual error in the east component mainly originated from the north attitude (roll) error. Thus, variance of north and east attitude error in the Kalman filter could be used as a weight in the optimal linear smoothing. Figure 18 shows the estimated variances of attitude error and the high frequency part of gravity disturbance horizontal components in line 4. As shown in Equations (10) and (11), if the variance is larger, the final result is less affected by corresponding results. The fusion results by the method are shown in Figure 19. The detail repeatability in 6 km resolution is shown in Table 5. After the application of the method, the repeatability of gravity disturbance horizontal components can reach the 1.83 mGal and 1.80 mGal under resolution of 6 km. Compared with the results shown in Figure 14, there are obvious improvements in the repeatability of both components and the clutter signal is also reduced, which prove the effectiveness of the accuracy improvement method.

## 5. Discussion

The effectiveness and practicability has been validated by an actual strapdown gravimetry test. In this section, WCF is compared with the optimal linear smoothing as an alternative data fusion method. The results of horizontal gravity disturbance in higher resolutions are also presented. Furthermore, the performance of the accuracy improvement method under the condition of the no gravity field model correction is discussed.

### 5.1. Apply Wavenumber Correlation Filter to Fuse the Results from Two Algorithms

The optimal linear smoothing is successfully applied to the combination of forward and backward algorithms. Another method for taking advantage of both forward and backward algorithms is wavenumber correlation filter (WCF) [10]. The basic hypothesis of the WCF is that the measurement results should be tightly correlated, while the systematic errors should be less correlated. The WCF decomposes time domain data into frequency bands using Fourier transformation, and then computes the correlation coefficients at the same frequency point that follows Equation (17):

(17) $$\begin{array}{c} CC_{k}=\cos\left(\theta_{k}\right)=\frac{F_{X}\left(k\right)\cdot F_{Y}\left(k\right)}{|F_{X}\left(k\right)||F_{Y}\left(k\right)|}, \end{array}$$

where $CC_{k}$ is the correlation coefficients at frequency point *k*, $F_{X}\left(k\right)$ is the Fourier transformation result of data series *X* at frequency point *k* and $F_{Y}\left(k\right)$ is the Fourier transformation result of data series *Y* at frequency point *k*.

Based on the correlation coefficients and a given threshold *T*, if the correlation coefficient is smaller than the threshold at certain frequency, the component at that frequency is considered to be the noise. In order to extract signals that are directly correlated, WCF filters only pass the frequency components, at which the $CC_{k}$ is larger than the threshold, and then it is eliminated according to Equation (18). Finally, the gravity signal reconstruction can be achieved by an inverse Fourier transform. When the WCF was applied in gravimetry, it usually fused data from two different repeat lines to reduce error, which is inefficient. Fortunately, combining the data from forward and backward algorithms can largely solve the problem of inefficiency:

(18) $$\begin{array}{c} F_{x,y}\left(k\right)=\left\{\begin{array}{cc} \frac{1}{2}\left(F_{X}\left(k\right)+F_{Y}\left(k\right)\right), & \mathrm{if}\;CC_{k}>T,\\ 0, & \mathrm{if}\;CC_{k}<T, \end{array}\right. \end{array}$$

where *T* is threshold and $F_{x,y}\left(k\right)$ is the frequency domain data after filtering.

**Figure 20:**
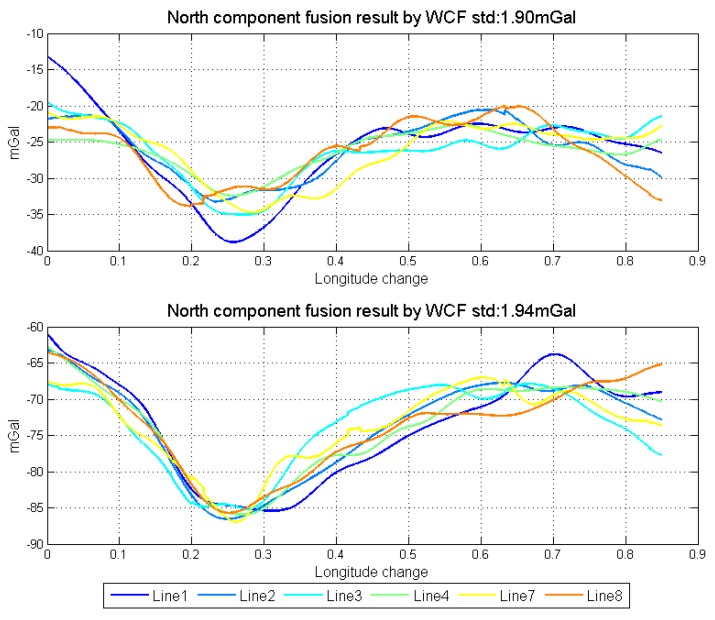
Data fusion results by WCF.

Fusing the data from forward and backward algorithms meets the requirement of WCF and can conquer the premise of repeat lines. When use WCF to eliminate the error, threshold selection is directly related to the performance of the filter. Figure 20 shows the results of using WCF to fuse the data, in which the threshold is given as −0.6. Comparing the two fusion methods, the optimal linear smoothing algorithm is better than WCF. It is because WCF is the average algorithm in the frequency domain after eliminating the weak correlated components, in which the characteristics of the data itself are also not considered.

### 5.2. The Result in Higher Resolution

**Figure 21:**
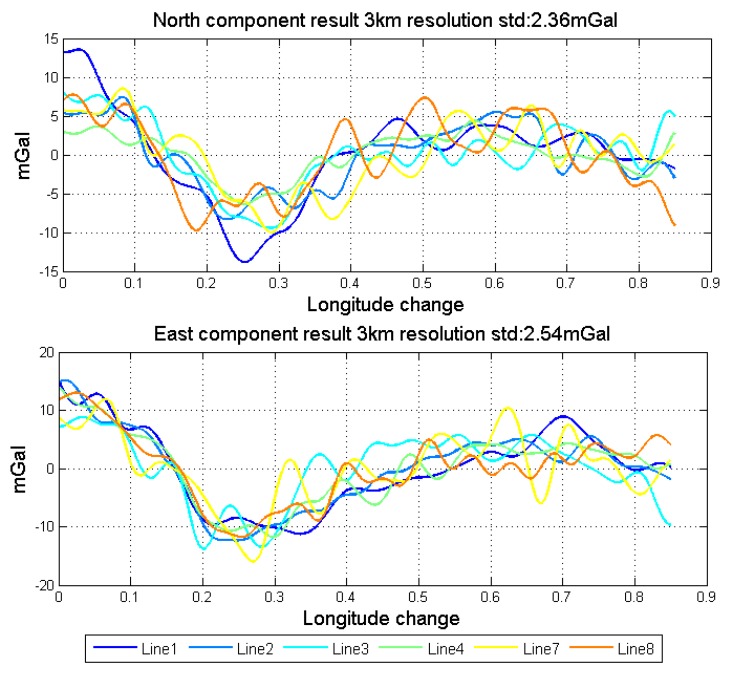
Horizontal components fusion result in 3 km resolution.

As mentioned, the vector gravimetry suffers more from the attitude error when compared with the scalar gravimetry. In the actual flight test, the repeatability of scalar results in normal lines could reach 1 mGal under the resolution of 3 km, while the repeatability of horizontal components of gravity disturbance vector results is about 1.8 mGal under the resolution of 6 km after data fusion. When we improve the resolution, the repeatability will reduce to some degree. Changing the cut-off frequency of the low pass filter, the repeatability of different resolutions are shown in Table 6. Figure 21 shows the results in 3 km resolution. Although the performance dose not decline much with the improvement of resolution, the noise part of the gravity disturbance vector signal significantly enhanced. This phenomenon is mainly caused by the insufficient attitude measurement accuracy. Thus, the essential way to improve the accuracy of vector gravimetry is to develop the proper algorithm, such as Refs. [18,19]. Another fundamental approach is to use the higher performance sensors, especially high precision gyroscopes, the bias stability of which should be better than 0.002 deg/h or even higher.

### 5.3. The Repeatability under the Absence of Gravity Field Information

The gravity field model has good accuracy in the low frequency part of the gravity disturbance vector, but the value from the gravity field model usually has a deviation when compared with the true value in a certain area. However, if the accuracy of the gravity field model is poor or the deviation between the gravity field model and true value is no longer constant, what is the performance of the method shown in the paper?

**Figure 22:**
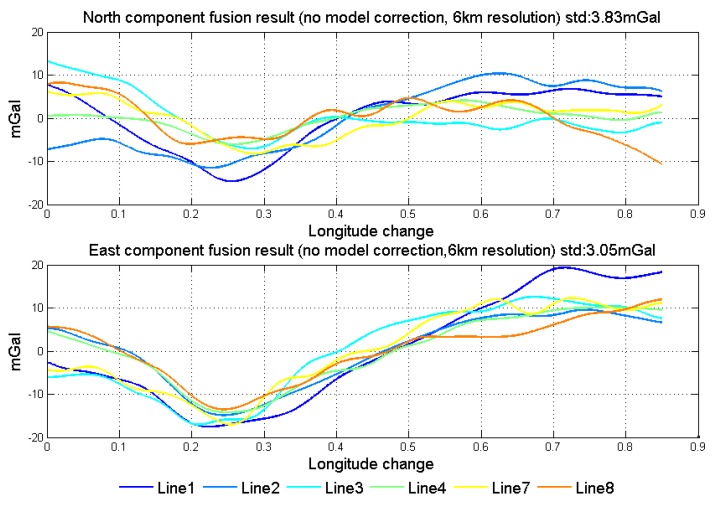
Results in 6 km resolution under the condition of no gravity field model correction.

We suppose that the base point information and method of end-matching could be applied in this kind of condition. Thus, after fusing the data from forward and backward algorithms, the mean value could be subtracted from the horizontal components (zero-mean transformation), the result of which is shown in Figure 22. Compared with the result shown in Figure 19, there is clear drift in the two horizontal components, which are mainly caused by the error in the low frequency part. Thus, we can conclude that accurate prior information and high stability accelerometers are very helpful to measure the true gravity field information.

In the following research, improvements in algorithms and hardware should be considered at the same time to comprehensively promote the performance of airborne vector gravimetry.

## 6. Conclusions

The research presented here developed an effective approach for strapdown gravimetry accuracy improvement. The advantages of the method can be summarized as follows:

1. The backward algorithm not only shows different characteristics in the standalone inertial calculation, but also has different convergence in the Kalman filter, which provides a backtracking result for the vector gravimetry.
2. Fusion of the data from forward and backward algorithms satisfies the precondition of error compensation and the repeat lines are not needed.
3. Based on an optimal linear filter, the data can be fused optimally rather than empirically.

The accuracy improvement method was given based on the promising simulation results. Applying to the data from an actual flight test, the improved gravity disturbance vector results can be obtained, especially for the horizontal components. The vertical components is 1 mGal under the resolution of 3 km. After applying the method, the north gravity disturbance component could reach the accuracy of 1.83 mGal and the east gravity disturbance component could reach the 1.80 mGal, both of which is under the resolution of 6 km. For considering the characteristics of the data itself, the optimal linear smoothing algorithm is better than WCF when fusing the results from two algorithms. Furthermore, the repeatability does not decline much with the improvement of resolution. To further test the method, the condition of no gravity field model correction is also presented, in which the method still shows good performance.

For further studies, the accuracy of the gravity disturbance vector obtained from the accuracy improvement method should be compared to the accurate gravity field control data. To fully achieve the goal of high accuracy airborne vector gravimetry, the excellent algorithm and high accuracy hardware system should be applied at the same time.

## Figures and Tables

**Table 1:** Error parameters in simulation.

Accelerometer	Noise	Bias (mGal)	Scale Factor Error
*x*-axis	10	100	20
*y*-axis	10	100	20
*z*-axis	10	100	10
Gyro	Noise (deg/h^1/2^)	Bias (deg/h)	
*x*-axis	0.003	0.03	
*y*-axis	0.003	0.03	
*z*-axis	0.003	0.03	
	Latitude (m)	Longitude (m)	Height (m)
GNSS position error	0.1	0.1	0.1
	North (m/s)	East (m/s)	Down (m/s)
GNSS velocity error	0.05	0.05	0.05

**Table 2:** Properties of SGA-WZ02.

Properties	Details
Static precision	0.5 mGal (24 h)
Max overload	4 g
Working temperature	−10–50 degree Celsius
Weight	45 kg
Dimension	460×350×580 mm
Stable power consumption	<150 W

**Table 3:** Characteristics of flight tests.

Characteristic	Details
Aircraft type	Cessna208
Atmosphere conditions	Smooth to turbulence
Altitude above standard ellipsoid	1500 m
Flight speed	60 m/s
Trace control method	Autopilot
Sampling frequency of GNSS	2 Hz
Sampling frequency of SINS	200 Hz

**Table 4:** Repeatability results of repeat lines (mGal).

		Line 1	Line 2	Line 3	Line 4	Line 7	Line 8	All
	North	24.56	42.00	12.02	7.24	11.87	4.90	21.32
Forward	East	9.33	3.29	2.18	3.46	5.45	3.82	5.15
	Down	0.91	1.25	0.75	1.05	0.81	1.11	1.00
	North	5.70	3.09	4.24	6.81	8.48	10.40	6.91
Backward	East	19.86	9.10	21.76	12.84	27.22	47.45	26.17
	Down	0.93	1.26	0.75	1.03	0.83	1.10	1.00

**Table 5:** Repeatability of data fusion results (mGal).

	Line 1	Line 2	Line 3	Line 4	Line 7	Line 8	All
North	2.18	1.03	1.60	1.70	1.87	1.98	1.83
East	2.02	0.94	2.71	0.95	1.55	1.61	1.80
Down	0.45	0.62	0.33	0.45	0.46	0.57	0.48

**Table 6:** Repeatability in different resolutions (mGal).

Resolution	Component	Line 1	Line 2	Line 3	Line 4	Line 7	Line 8	All
3 km	North	2.48	1.60	2.05	1.86	2.52	2.85	2.36
	East	2.38	1.53	3.39	1.50	3.18	2.12	2.54
	Down	0.79	0.83	0.63	0.94	0.81	0.93	0.85
4.2 km	North	2.33	1.26	1.79	1.77	2.12	2.34	2.05
	East	2.20	1.18	2.95	1.15	2.23	1.81	2.10
	Down	0.54	0.68	0.39	0.68	0.61	0.66	0.60
5.4 km	North	2.24	1.10	1.62	1.74	1.93	2.09	1.90
	East	2.13	1.01	2.75	0.97	1.71	1.69	1.90
	Down	0.47	0.63	0.34	0.51	0.50	0.59	0.51

## Appendix A

The details of the sub-matrix in Equation (8) is shown as follows:

(A1) $$PP=\left[\begin{array}{ccc} 0 & 0 & \frac{-V_{n}}{{\left(R_{M}+H\right)}^{2}}\\ \frac{V_{E}tanL}{(R_{N}+H)cosL} & 0 & \frac{-V_{E}}{{\left(R_{N}+H\right)}^{2}cosL}\\ 0 & 0 & 0 \end{array}\right],$$

(A2) $$PV=\left[\begin{array}{ccc} \frac{1}{R_{M}+H} & 0 & 0\\ 0 & \frac{1}{(R_{N}+H)cosL} & 0\\ 0 & 0 & -1 \end{array}\right],$$

(A3) $$PA=\left[\begin{array}{ccc} 0 & 0 & 0\\ 0 & 0 & 0\\ 0 & 0 & 0 \end{array}\right],$$

(A4) $$VP=\left[\begin{array}{ccc} -V_{E}\left(2\omega_{ie}^{n}cosL+\frac{V_{E}sec^{2}L}{R_{N}+H}\right) & 0 & \left(\frac{-V_{N}V_{D}}{{\left(R_{M}+H\right)}^{2}}+\frac{V_{E}^{2}tanL}{{\left(R_{N}+H\right)}^{2}}\right)\\ 2\omega_{ie}^{n}\left(V_{N}cosL-V_{D}sinL\right)+\frac{V_{E}V_{N}sec^{2}L}{R_{N}+H}) & 0 & \left(\frac{-V_{E}V_{D}}{{\left(R_{N}+H\right)}^{2}}-\frac{V_{E}V_{N}tanL}{{\left(R_{N}+H\right)}^{2}}\right)\\ 2\omega_{n}^{ie}V_{E}sinL & 0 & \left(\frac{V_{E}^{2}}{{\left(R_{N}+H\right)}^{2}}+\frac{V_{N}^{2}}{{\left(R_{M}+H\right)}^{2}}\right) \end{array}\right],$$

(A5) $$VV=\left[\begin{array}{ccc} \frac{V_{D}}{R_{M}+H} & -2(\omega_{ie}^{n}sinL-\frac{V_{E}tanL}{R_{N}+H}) & \frac{V_{N}}{R_{M}+H}\\ \omega_{ie}^{n}sinL+\frac{V_{E}tanL}{R_{N}+H} & \frac{V_{D}}{R_{N}+H}+\frac{V_{N}tanL}{R_{N}+H} & 2\omega_{ie}^{n}cosL+\frac{V_{E}}{R_{N}+H}\\ \frac{-2V_{N}}{R_{M}+H} & -2(\omega_{ie}^{n}cosL-\frac{V_{E}}{R_{N}+H}) & 0 \end{array}\right],$$

(A6) $$VA=\left[\begin{array}{ccc} 0 & -f_{D} & f_{E}\\ f_{D} & 0 & f_{N}\\ -f_{E} & -f_{N} & 0 \end{array}\right],$$

(A7) $$AP=\left[\begin{array}{ccc} -\omega_{ie}^{n}sinL & 0 & \frac{-V_{E}}{{\left(R_{N}+H\right)}^{2}}\\ 0 & 0 & \frac{V_{N}}{{\left(R_{M}+H\right)}^{2}}\\ -\omega_{ie}^{n}cosL-\frac{V_{E}}{\left(R_{N}+H\right)cos^{2}L} & 0 & \frac{V_{E}tanL}{{\left(R_{N}+H\right)}^{2}} \end{array}\right],$$

(A8) $$AV=\left[\begin{array}{ccc} 0 & \frac{1}{\left(R_{N}+H\right)} & 0\\ -\frac{1}{\left(R_{N}+H\right)} & 0 & 0\\ 0 & -\frac{tanL}{\left(R_{N}+H\right)} & 0 \end{array}\right],$$

(A9) $$AA=\left[\begin{array}{ccc} 0 & -\omega_{ie}^{n}sinL-\frac{V_{E}tanL}{\left(R_{N}+H\right)} & \frac{V_{N}}{\left(R_{M}+H\right)}\\ \omega_{ie}^{n}sinL+\frac{V_{E}tanL}{\left(R_{N}+H\right)} & 0 & \omega_{ie}^{n}cosL+\frac{V_{E}}{\left(R_{N}+H\right)}\\ -\frac{V_{N}}{\left(R_{M}+H\right)} & -\omega_{ie}^{n}cosL-\frac{V_{E}}{\left(R_{N}+H\right)} & 0 \end{array}\right],$$

where *L* is the latitude, *H* is the height, $R_{M}$ is the meridian radius of earth and $R_{N}$ is the prime circle radius of earth.
